# Chloroform Inhalations in Strychnia Poisoning

**Published:** 1869-06

**Authors:** 


Chloroform Inhalations in Strychnia Poisoning.—A number of our exchanges report cases of poisoning from strychnia successfully treated by the inhalation of chloroform. In several instances the quantity of poison swallowed was quite enough to cause death, and the inhalations were continued twenty-four hours. Should further trial confirm these results, chloroform will speedily take the place of all other antidotes of strychnia.

